# Placenta-Specific Protein 1: A Potential Key to Many Oncofetal-Placental OB/GYN Research Questions

**DOI:** 10.1155/2014/678984

**Published:** 2014-03-17

**Authors:** Eric J. Devor, Henry D. Reyes, Donna A. Santillan, Mark K. Santillan, Chinenye Onukwugha, Michael J. Goodheart, Kimberly K. Leslie

**Affiliations:** ^1^Department of Obstetrics and Gynecology, University of Iowa Carver College of Medicine, 3234 MERF, Iowa City, IA 52242, USA; ^2^Lincoln University, Lincoln University, Pennsylvania, PA 19352, USA; ^3^Holden Comprehensive Cancer Center, University of Iowa Hospitals and Clinics, Iowa City, IA 52242, USA

## Abstract

Placenta-specific protein 1 (PLAC1) is a secreted protein found in trophoblasts. Several reports implicate a central role for PLAC1 in establishment and maintenance of the placenta. In addition to placentae PLAC1 is expressed in a variety of solids including breast, endometrial, and ovarian cancers. In order to show that PLAC1 is potentially relevant to a number of research questions in OB/GYN, we report on PLAC1 expression in a selected panel that includes two choriocarcinoma cell lines, normal placental tissues, and endometrial and ovarian tumors. We report for the first time that PLAC1 is also expressed in human fetal tissues. PLAC1 is transcriptionally heterogeneous with one promoter (P1) generating two transcripts with alternately spliced 5' UTRs and the other promoter (P2) generating a third transcript. Placental tissues favor P2 transcripts, while P1 is favored in most of the other cells. Mechanisms determining multiple PLAC1 transcripts and promoter preferences are as yet unknown, but it is clear that this protein is likely to be important in a variety of phenomena relevant to both gynecologic oncology and maternal-fetal medicine.

## 1. Introduction

Placenta-specific protein 1 (PLAC1), encoded on human chromosome Xq26, is a small (212 amino acid) secreted protein whose expression was, until recently, believed to be exclusively limited to placental trophoblasts [1, 2]. The importance of PLAC1 to the establishment and maintenance of normal gestation has been amply demonstrated through the generation of a* Plac1 *knockout mouse model in which placentae exhibit placentomegaly and pups show numerous phenotypes consistent with intrauterine growth restriction (IUGR) [3]. Several reports over the past few years have demonstrated PLAC1 expression in a variety of human solid tumors including lung cancers [4], breast cancers [5], hepatocellular and colorectal cancers [6, 7], gastric cancers [8], and uterine cancers [9]. In addition, PLAC1 expression has been reported in nearly one hundred human cancer cultured cell lines representing fourteen different cancers [4–6, 9]. Because of this additional expression milieu PLAC1 has been described as an oncoplacental protein [10]. Recently,* Plac1 *expression was reported in* fetal* mouse tissues by both* in situ* hybridization and qPCR [11]. Thus, PLAC1 can now be described as the first oncofetal-placental protein.

The* PLAC1* gene is composed of six exons spanning nearly 200 kilobases (kb), but the entire 639 bp coding sequence is contained within the 898 bp long exon 6. The five upstream exons make up a set of alternatively spliced 5′ UTRs transcribed off of two promoters. Promoter 1, described by Chen et al. [12], lies upstream of exon 1 and produces two transcripts; we have designated P1Long and P1Short. Promoter 2, described both by Chen et al. [12] and Koslowski et al. [13], lies upstream of exon 4 and produces a single P2 transcript. Several studies have suggested that placental tissues favor Promoter 2 and tumors predominantly favor Promoter 1 [9, 12–14]. We here confirm this suggestion in a tissue panel composed of six placentas, six endometrial adenocarcinomas, and six serous ovarian carcinomas. In addition, we have included two placenta-derived uterine choriocarcinoma cell lines (JEG-3 and JAR) which, despite arising from placental trophoblasts, utilize Promoter 1 like other cancers. Finally, we confirm PLAC1 expression in fetal tissues through qPCR amplification of PLAC1 message in human fetal brain, heart, liver, and kidney, each of which favors Promoter 1 transcription.

Taken together, our data and those produced by the studies cited above strongly suggest that PLAC1 should be considered a prime object of study in both gynecologic cancers and gestational disorders such as preeclampsia and preterm labor.

## 2. Materials and Methods

### 2.1. Tissues and Cells

Choriocarcinoma cell lines JEG-3 and JAR were obtained from the American Type Culture Collection (ATCC). Placental tissues were selected from the Institutional Review Board approved Maternal Fetal Tissue Bank (MFTB) maintained in the Department of Obstetrics and Gynecology. The six tissues were all from uncomplicated, term pregnancies (average 39.4 weeks). Three were spontaneous vaginal deliveries with normal labor and three were Cesarean section deliveries. Core sections of placenta were stored in RNAlater (Life Technologies) at −80°C. Both the endometrial and ovarian carcinomas were collected under informed consent and Institutional Review Board approval from patients undergoing surgery at the University of Iowa Hospitals and Clinics. Three of the endometrial cancers were early stage endometrioid adenocarcinomas and three were Stage III serous adenocarcinomas. All six ovarian cancers were Stage III serous adenocarcinomas.

Enrollment into any of the approved banks in the Department of Obstetrics and Gynecology allows access to relevant clinical data contained within the patient medical records.

### 2.2. Cell Culture and RNA Purification

JEG-3 and JAR cells were cultured under optimal conditions (JEG-3 in EMEM 10% FBS; JAR in RPMI 1640 10% FBS) and cells for RNA purification were harvested at 80–90% confluence. Placental tissues and endometrial and ovarian tumors were removed from −80°C storage and transitioned in RNAlaterICE (Life Technologies) for 24 to 48 hours prior to RNA extraction. All RNA purifications were performed with the miRvana RNA extraction kit (Life Technologies) according to manufacturers' recommendations. RNA yield and purity were assessed on a NanoDrop Model M-1000 spectrophotometer and an Agilent Model 2100 Bioanalyzer. Only RNAs with an integrity number (RIN) [15] of at least 5.0 (range 5.2 to 9.7) were used in this study.

Human fetal tissue total RNAs from brain, heart, liver, and kidney were purchased from Clontech.

### 2.3. Quantitative PCR

Equal aliquots of total RNA from each sample (250 ng) were reverse transcribed using SuperScript III reverse transcriptase with oligo-dT priming (Life Technologies). SYBR Green qPCR was carried out in Power SYBR Green (Life Technologies) using primers designed to amplify total PLAC1 message as well as Promoter 1-specific and Promoter 2-specific messages as previously reported [9]. We also generated a series of* PLAC1 *plasmids from cDNA for the purpose of being used in standard curve calculations for Promoter 1, Promoter 2, and Total PLAC1 expression. Total and promoter-specific standard curve amplifications were carried out against Promoter 1- and Promoter 2-transcript clones standardized to a range of 10^12^ to 10^6^ copies. Primer efficiencies estimated from the standard curves are 0.968 for total PLAC1 primers, 0.946 for Promoter 1-specific primers, and 1.023 for Promoter 2-specific primers. All qPCR amplifications were performed on an Applied Biosystems Model 7900 Genetic Analyzer in the University of Iowa DNA Core Facility.

Absolute primer-specific qPCR amplification values were calculated against the standard curves via linear regression. Relative within-group comparisons used cycle threshold values (Ct) from qPCR normalized against 18S rRNA. Fold change was then determined via the conventional ΔΔCt method [16, 17]. Statistical significance was evaluated using a standard* t*-test with unequal variances [18].

### 2.4. p53 Sequencing

Mutation status of the p53 oncogene in the cancer samples was determined by direct sequencing of p53 cDNAs obtained from SuperScript III oligo-dT primed reverse transcription. PCR primers p53For: 5′-CATGTGCTCAAGACTGGCGCTAAA-3′ (*T*_*m*_ = 59.8°C) and p53Rev: 5′-AATGGAAGTCCTGGGTGCTTCTGA-3′ (*T*_*m*_ = 60.3°C) produce an 1182 bp amplicon containing the entire p53 coding region. These primers and those used for qPCR were all purchased from Integrated DNA Technologies (IDT) after PrimerQuest assessment of secondary structure formation.

## 3. Results

**Figure 1:**
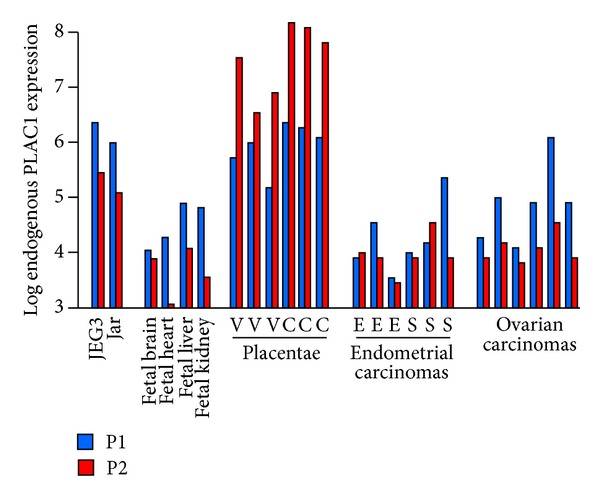
Log_10_ PLAC1 expression in all tissues and cells used in this study. Expression is shown by promoter based upon primer pair-specific standard curve regressions. All values are single samples averaged from three technical replicates. In placentae, V is a normal, spontaneous labor vaginal delivery and C is Cesarean section. In the endometrial carcinomas, E is endometrioid adenocarcinoma and S is serous adenocarcinoma. All ovarian tumors are serous histology.

Promoter-specific PLAC1 expression levels in the two choriocarcinoma cell lines as well as in fetal tissues, placentae, and the two gynecologic cancers are shown in Figure 1. Placental tissues display considerably more PLAC1 expression than do tumors and the Promoter 2 preference in the placental tissues is evident. As expected from a number of studies, Promoter 1 is favored in ten of twelve tumors though ovarian tumors are more consistent than are endometrial tumors. Our previous observation [9] that endometrial serous adenocarcinomas have greater PLAC1 expression levels than do endometrial endometrioid adenocarcinomas is supported in this much smaller panel with the three serous tumors exhibiting an 8.5-fold higher PLAC1 expression than the three endometrioid tumors (*P* = 0.07). Those same endometrial serous tumors display no difference in average PLAC1 expression compared with the serous ovarian tumors (1.19-fold higher, *P* = 0.86). This observation is also consistent with our suggestion that more advanced stage tumors have higher PLAC1 expression than do less advanced tumors [9].

Inclusion criteria for placental tissue selection for this panel required that the birth be full term and medically unremarkable. Both the much higher levels of PLAC1 expression and the clear predominance of Promoter 2 transcripts are evident (Figure 1). We chose three samples from patients who had spontaneous vaginal deliveries and three from patients who had Caesarian section deliveries with all other critical covariates, such as age and BMI, being similar. We find that the labored vaginal group displays a 2.5-fold decrease in PLAC1 expression compared with the unlabored Caesarian section group (*P* = 0.07). While a larger sample is obviously required, this observation is consistent with the view that PLAC1 levels respond to the onset of labor [19, 20].

Choriocarcinoma cells have much higher PLAC1 expression levels than do the tumors. This, of course, may be attributable to the fact that these cells are homogeneous, were grown under ideal conditions in a laboratory, and were harvested well before any potential crowding effects might be elicited. Again, a clear preference for Promoter 1 transcripts is evident.

The recent report of PLAC1 expression in fetal mouse tissues [11] prompted us to add a human fetal tissue RNA panel. Our results confirm those reported in mouse fetal tissues and extend them by demonstrating that Promoter 1 transcripts are favored in these tissues as well. Thus, to date, with the exception of a few solid tumors, only the placenta displays a predominance of Promoter 2 transcripts.

**Figure 2:**
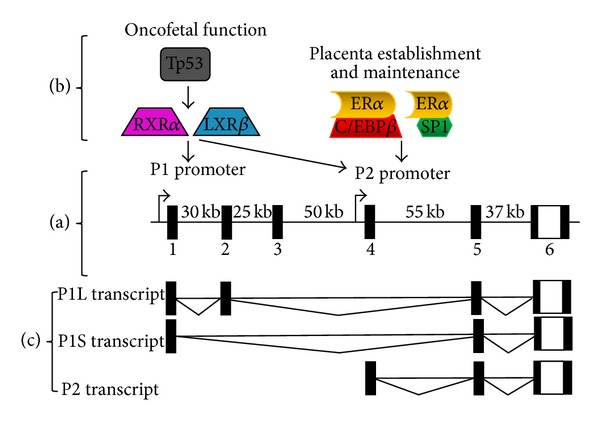
Human PLAC1 gene organization and transcription. (a) The genomic structure of the PLAC1 gene and the location of the two promoters are shown. Noncoding DNA is indicated by the filled boxes while the 3′-most part of the 5′ UTR, the complete coding region, and the 3′ UTR are all contained in exon 6. The distances between the exons are also shown. (b) Transcription factors and suspected transcription influencing factors associated with the two PLAC1 promoters. (c) mRNA transcripts produced by the human PLAC1 gene that have been cloned by the authors. All three transcripts, P1L (ong), P1S (hort), and P2, have been cloned from a single reverse transcription in several instances including both endometrial and ovarian tumors.

In the course of cloning total and promoter-specific PLAC1 plasmids we detected one sequence for Promoter 2-specific clones, but there were two different sequences among the Promoter 1-specific clones. Promoter 2 clones were all composed of exons 4-5-6 as expected (Figure 2), but Promoter 1 clones contained a mixture of sequences composed of exons 1-2-5-6 (1144 bp) and 1–5-6 (1059 bp). We have termed these latter sequences Promoter 1L (ong) and Promoter 1S (hort) and have detected both transcripts in several tumors along with Promoter 2 transcripts. No mechanism accounting for this transcriptional heterogeneity has been offered to date.

## 4. Discussion

Since its discovery just over a decade ago [1], PLAC1 has slowly emerged as far more than a simple placenta-specific protein. Its importance in the establishment and maintenance of the placenta has been well documented [2, 3, 21] as has its potential involvement in both normal and abnormal gestation [19, 20, 22–24]. There is also evidence that high levels of anti-PLAC1 antibody may be involved in some cases of unexplained infertility [25, 26]. In addition, as noted above, PLAC1 expression has been detected in a number of human cancers and cancer cell lines. In one series of experiments with MCF7 breast cancer cells, RNAi-mediated silencing of PLAC1 succeeded in significantly reducing migration and invasion capabilities and almost completely ablating proliferation [5]. These expression characteristics in placentae and in solid tumors earned PLAC1 the designation of oncoplacental protein [10]. Very recently, however, PLAC1 expression has been reliably detected in mouse fetal tissues [11] and, here, such detection has been extended to include human fetal tissues. Thus, PLAC1 must now be considered to be an oncofetal-placental protein.

Oncofetal-placental PLAC1 expression is one part of the story, but the PLAC1 gene itself displays an unusual characteristic. The six exons of the PLAC1 gene span nearly 200 kb of chromosome Xq26.3, but its entire coding region and 3′ UTR is contained within the sixth and largest codon (Figure 2). The remaining five exons comprise a series of 5′ UTRs all of which end with exons 5 and 6. The composition of the 5′ UTR is determined by mRNA transcription from two promoters (Figure 2). Promoter 2 lies just upstream from exon 4, produces an mRNA composed of exons 4-5-6, and has been shown to be under the control of both an SP1 transcription factor and a rare isoform of CCAAT/Enhancer-binding protein *β* [13]. There is some additional evidence that Promoter 2 is trans-activated by estrogen receptor *α* (ER*α*) via a noncanonical pathway [13]. Promoter 1 was discovered more than 100 kb upstream from Promoter 2 just 5′ of the first of three previously unknown exons [27]. Transcription factors RXR*α* and LXR activate both Promoter 1 and Promoter 2, but it is Promoter 1 that is responsible for the majority of PLAC1 transcripts in both tumors and fetal tissues. It has recently been suggested that the oncogene Tp53 is involved in PLAC1 expression in that mutant Tp53 results in Promoter 1 activation [27]. Although our sample size is small and was not assembled to explore this question, we sequenced p53 in all twelve tumors. We found six mutant p53 genes, two of the three endometrial serous tumors, and four of the six ovarian tumors. There was a fourfold higher average PLAC1 expression among six tumors with mutant p53 genes than among the six with wild-type p53 genes. Though not statistically significant (*P* = 0.12), this trend is consistent with the idea that mutant Tp53 does increase PLAC1 expression through Promoter 1.

The current state of PLAC1 transcriptional relationships is summarized in Figure 2. It is evident that there are at least three PLAC1 mRNAs that differ by promoter usage and the composition of the 5′ UTR and that these transcripts are differentially represented in tumors, placentae, and fetuses. It is also clear that, since the PLAC1 protein coding region is contained within a single downstream exon, the effect of different promoters and alternatively spliced 5′ UTRs on PLAC1 protein production must be rigorously investigated. More functional studies centered on the mature PLAC1 protein and its role in placental and fetal development and maintenance as well as in gynecologic cancer origin and growth must also be undertaken. Thus, the apparent complexity of PLAC1 protein production, especially the relationships between genomic and proteomic characteristics, and the suspected role of this protein in fetal tissue development, in both normal and abnormal placentation and gestation and in gynecologic malignancies, makes it an important subject for women's health research going forward.
